# Fluency Expresses Implicit Knowledge of Tonal Symmetry

**DOI:** 10.3389/fpsyg.2016.00057

**Published:** 2016-02-03

**Authors:** Xiaoli Ling, Fengying Li, Fuqiang Qiao, Xiuyan Guo, Zoltan Dienes

**Affiliations:** ^1^School of Psychology and Cognitive Science, East China Normal UniversityShanghai, China; ^2^Department of Psychology, Zhejiang Normal UniversityJinhua, China; ^3^School of Education and Psychology, University of JinanJinan, China; ^4^Shanghai Key Laboratory of Magnetic Resonance, School of Psychology and Cognitive Science, East China Normal UniversityShanghai, China; ^5^Key Laboratory of Brain Functional Genomics, Ministry of Education, Shanghai Key Laboratory of Brain Functional Genomics, East China Normal UniversityShanghai, China; ^6^School of Psychology, Sackler Centre for Consciousness Science, University of SussexBrighton, UK

**Keywords:** fluency, implicit learning, symmetry, cross serial dependency, chunks

## Abstract

The purposes of the present study were twofold. First, we sought to establish whether tonal symmetry produces processing fluency. Second, we sought to explore whether symmetry and chunk strength express themselves differently in fluency, as an indication of different mechanisms being involved for sub- and supra-finite state processing. Across two experiments, participants were asked to listen to and memorize artificial poetry showing a mirror symmetry (an inversion, i.e., a type of cross serial dependency); after this training phase, people completed a four-choice RT task in which they were presented with new artificial poetry. Participants were required to identify the stimulus displayed. We found that symmetry sped up responding to the second half of strings, indicating a fluency effect. Furthermore, there was a dissociation between fluency effects arising from symmetry vs. chunk strength, with stronger fluency effects for symmetry rather than chunks in the second half of strings. Taken together, we conjecture a divide between finite state and supra-finite state mechanisms in learning grammatical sequences.

## Introduction

Implicit learning refers to the process by which people acquire unconscious knowledge of the structure of the environment (e.g., Reber, 1967, 2013; Williams, 2009; Rebuschat, 2013). Two central questions for the field are what sort of structures can be implicitly learnt (e.g., Pothos, 2007; Remillard, 2011; Fitch and Friederici, 2012), and by what computational mechanisms (e.g., Cleeremans and Dienes, 2008; Chubala and Jamieson, 2013)? The questions are of course related in that certain structures require learning devices of a certain level of complexity; and a given learning device defines a class of learnable structures. One prominent way of classifying structure complexity that may be relevant to implicit learning is whether the structure could be processed by a device of finite state or rather more than finite state complexity (e.g., Poletiek, 2011; Rohrmeier et al., 2014).

For example, learning chunks (Servan-Schreiber and Anderson, 1990; Rohrmeier and Rebuschat, 2012) or fixed repetition patterns (Vokey and Brooks, 1992; Tunney and Altmann, 2001) requires no more than finite state capacity; conversely, learning symmetries (Westphal-Fitch et al., 2012), center embeddings or cross serial dependencies (e.g., de Vries et al., 2012) requires supra-finite state capacity, if those structures have been learnt as such. Indeed, Friederici et al. (2006) suggested that different mechanisms and brain regions are involved in learning (natural or artificial) grammatical structure that divides precisely along the finite vs. supra-finite state boundary. We will explore the implicit learning of structures that were generated by grammars that lay either side of this boundary in order to explore the possible mechanism by which different structures are learnt.

Implicit learning is often explored with the artificial grammar learning (AGL) paradigm. In a typical AGL experiment, participants are exposed to letter strings generated by a complex set of rules, but they are not at this stage informed about the existence of rules. At test, participants are finally told that all the strings they encountered obeyed rules. They then judge whether new strings are grammatical or ungrammatical. The typical result shows that participants’ classification performance is above-chance even without awareness of rules (e.g., Reber, 1967, 1989) or recollections of particular exemplars (Dienes and Scott, 2005), suggesting that people can acquire some implicit knowledge about the underlying structure.

What structure can people implicitly learn? There is agreement that in AGL paradigms people can learn chunks (such as especially bigrams and trigrams that appeared in the string; e.g., the string XXRVTM includes the chunks XX, XR, RV, VT, TM, XXR, XRV, RVT, and VTM; see Perruchet and Pacteau, 1990; Knowlton and Squire, 1996), fixed repetition patterns (e.g., Mathews and Roussel, 1997; Lotz and Kinder, 2006), and specific exemplars (e.g., Higham, 1997; Jamieson and Mewhort, 2011). These structures can all be learnt by a device of finite state or lower capability, i.e., which only needs to learn a set of state transitions, but does not need to use a buffer to otherwise process the stimulus. Not all structures can be learnt by such a device. For example, learning to see a symmetry as a symmetry requires a working buffer in order to extract the hierarchical structure that defines the symmetry (e.g., Dienes and Longuet-Higgins, 2004; Dienes et al., 2011). People can quickly see mirror symmetries under many conditions (Tyler, 2002), indicating the relevance of symmetry to conscious perception (at least in the visual case). But can people implicitly learn to detect symmetry as a structure defining a set of stimuli?

Kuhn and Dienes (2005) found evidence that people were sensitive to a symmetry in sequences of musical notes. Each sequence consisted of an initial four notes stating a theme, followed by its musical inversion (produced by placing a horizontal mirror under the music score representing the theme). The resulting structure is formally identical to a cross serial dependency in language. People were more sensitive to the inversion symmetry in their liking ratings than in their rule classifications, providing evidence that the knowledge was unconscious. The material controlled chunk strength for inversions vs. non-inversions, ruling out an explanation in terms of chunk learning. Nonetheless, stimuli constructed according to any regularity will always instantiate a number of correlated regularities (Dulany, 1962). Thus it is important in concluding that implicit learning can occur for a new type of structure to control for structures already known to be implicitly learnable, and also to construct new stimuli in different ways to ensure conceptual replication.

**FIGURE 1:**
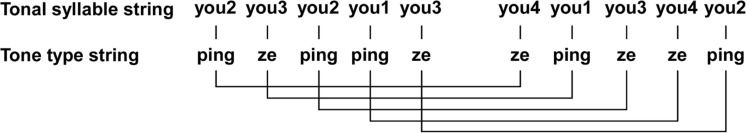
**An example of grammatical strings.** As in Jiang et al. (2012), the symmetry used in the current study was defined over the tones (with respect to the four tone types, where tones 1 and 2 are traditionally categorized as ping tones, while tones 3 and 4 are categorized as ze tones) with which Chinese syllables were spoken. Specifically, each string consisted of 10 tonal syllables and the tone type (pings or zes) of the previous five syllables predicted the following inversion, e.g., if the tone type of the first syllable was ping, then the tone type of the sixth syllable was ze, and if the type of the second syllable was ping, the tone type of the seventh syllable was ze, and so on. The inversion relation can be construed as an element to element mapping as shown: or as an operation on the whole of the first half which is then concatenated on the end. That is, if V represents the first half, –V is the inversion concatenated on (illustrating how symmetry is an “operation over variables”), transforming between pings and zes.

Jiang et al. (2012) investigated the implicit learning of inversions in a new paradigm, controlling a number of possible confounds, including both chunks and repetition structures. The symmetry rule employed by Jiang et al. (2012; and in the present research) was derived from the use of Chinese tones in Tang dynasty poetry. Jiang et al. (2012) constructed poems in which the sequence of tones in the last half was the mirror inversion of the tone sequence in the first half (the nature of the mirror inversion of the tone sequence can be seen in **Figure 1**). After repeating out loud such poems, participants could classify new poems with above chance accuracy, even when they said they were responding randomly or had based their answers on intuition with no idea of why they were right. That is, people seemed to acquire implicit knowledge of the presence of a type of symmetry.

Li et al. (2013) replicated the procedure but using a retrograde (i.e., formed by placing a vertical mirror at the end of a music score representing the theme; a center embedding) instead of an inversion (cross serial dependency), and also found implicit learning of the retrograde symmetry. Distinctively implicit learning of supra-finite state structures with rather different materials has also been argued for by Rohrmeier et al. (2012), Uddén et al. (2012), and Tanaka and Watanabe (2013, 2014). Orgs et al. (2013) argued that incidental exposure to symmetries influences aesthetic judgments, which may depend on implicit knowledge (Zizak and Reber, 2004). Further, de Vries et al. (2012) showed incidental learning of center embedding and cross-serial dependencies could occur, while making no claims as to the implicit nature of the knowledge. Other authors (e.g., Lai and Poletiek, 2011) have found intentional learning of similar structures.

Despite such evidence for the learnability of supra-finite state structures, the claim that the distinctively implicit learning of symmetry can occur is not settled. Kuhn and Dienes (2008) showed how a simple recurrent network (the SRN) could learn the materials of Kuhn and Dienes (2005) by learning fixed long-distance associations spanning four tones. Similarly, the test materials of Jiang et al. (2012) could be discriminated by knowledge of long-distance associations alone, spanning six tones (see Pacton and Perruchet, 2008, and Remillard, 2010, for the implicit learning of long distance associations). We will not in this paper directly address the issue of what has been learnt but rather consider contrasting predictions from two theories concerning the mechanism by which knowledge expresses itself; specifically, whether fluency plays a key role in the expression of the knowledge. We will now argue that the theory that people have learnt symmetry predicts a key role of fluency in the expression and use of that knowledge; conversely, the theory that people have learnt associations in the same way as occurs in AGL for finite state grammars predicts no role of fluency.

### The Role of Fluency

An indirect way of tackling the question of whether a symmetry has genuinely been learned is to consider whether symmetry learning should have properties different from associative learning. A large body of research has shown that human prefer symmetry than asymmetry (e.g., Gangestad et al., 1994; Humphrey, 1997; Rhodes et al., 1998, 1999; Reber and Schwarz, 2006). One prominent account for this robust phenomenon is that symmetry might facilitate processing fluency (Reber, 2003; Reber et al., 2004; Winkielman et al., 2006), i.e., the speed with which processing comes to completion. Van der Helm and Leeuwenberg (1996) argued that symmetrical regularities are detected faster and discriminated more easily. To detect symmetry is, by definition, to find an invariant: That which is preserved across the different symmetric instantiations. Thus, detecting symmetry should allow for compression, faster encoding and easier storage of information. Indeed, Enquist and Arak (1994) used computer simulation to show that the visual system processes symmetrical patterns especially efficiently (cf. Enquist and Johnstone, 1997).

Processing symmetry in auditory sequences presents a different computational problem from the visual case; nonetheless, symmetry still in principle allows informational compression. While not all compression may lead to fluency (Seger, 1997), in the visual case symmetries are indeed processed rapidly (Wagemans, 1995). These results, in conjunction with previous findings showing that fluency is closely connected with positive affective response (e.g., Reber et al., 1998; Winkielman and Cacioppo, 2001) may explain why people preferred the grammatical rather than ungrammatical auditory sequences in Kuhn and Dienes’ (2005), Jiang et al. (2012), and Li et al. (2013): symmetry produced fluency, and fluent experiences were preferred. The hypothesis that fluency enhances preference toward grammatical auditory sequences was directly tested by Qiao et al. (in preparation). In their Experiment 1, the inter-stimulus interval (ISI) between each auditory syllable of a string (which varied in Chinese tones) was manipulated (from 80 to 120 ms). They found that syllable strings with shorter ISI were judged more positively. Having established a workable method of inducing fluency, Experiment 2 employed the same manipulation after training on the inversion. In the test phase people were asked to classify sequences as grammatical or not. They found that fluency (as manipulated by ISI) could also affect grammaticality judgments (which Kinder et al., 2003, and Scott and Dienes, 2010a found did occur for visual material if it disappeared as soon as it was completely displayed).

Fluency is also a possible explanation of preferences in other AGL studies using finite state grammars (Gordon and Holyoak, 1983; Manza et al., 1998; Newell and Bright, 2001), possibly associated with the use of fringe feelings (Norman et al., 2006, 2010). To test such a claim directly, Buchner (1994) exposed participants to grammatical strings in training and presented strings with a perceptual clarification procedure in test. He concluded that there was no systematic relation between speed of identification and grammaticality judgments. In contrast, Kinder et al. (2003) experimentally manipulated fluency by varying the rate at which strings were revealed during the perceptual clarification task, and found people were sensitive to fluency in AGL judgments. Scott and Dienes (2010a) replicated these results, showing that faster clarifying strings were more often endorsed as grammatical – but only where exposure was brief, i.e., strings were not available for reference while making grammaticality judgments. That is, under typical conditions of AGL experiments where participants make decisions in their own time, fluency did not influence grammaticality judgments. Johansson (2009) similarly found that fluency was only used in AGL when response deadlines were short. Scott and Dienes (2010b) further showed that the grammaticality of the strings themselves was not expressed in processing fluency. Scott and Dienes (2010b) replicated the lack of relation between grammaticality and fluency in a transfer paradigm, where the same grammar was presented in a different perceptual domain.

However, we cannot conclude that fluency plays no role in implicitly learning to detect chunks. Chunk strength can lead to fast responding, as shown by the sequential reaction time (RT) paradigm (e.g., Brown et al., 2010; Janacsek et al., 2012; Sanchez and Reber, 2013, for examples). Indeed, Shanks and Berry (2012) argued that in any memorial situation there is only one source of evidence stored, which can express itself as fluency or as judgments (e.g., recognition judgments, well-formedness judgments, etc.) (contrast, e.g., Rünger et al., 2009). But the Shanks and Berry (2012) model does not postulate that judgments are based on fluency; instead, both fluency and judgments are based on a third factor, represented evidence. In a given situation the evidence may, for example, express itself very weakly as fluency and strongly as judgments – or vice versa.

Seger (1997) argued that the SRT task demonstrates a special motor-linked form of implicit learning, but independent of such knowledge there is a judgment-linked form of implicit learning, as shown in standard AGL. In the case of SRT, the training typically consists of learning to make the same motor responses (or attentional movements) as participants are tested on (and where it does not, learning effects are considerably reduced: e.g., Gheysen et al., 2009; cf. Guo et al., 2013). Thus, we conjecture that when people implicitly learn to perceive that stimuli have a certain structure (rather than learn to make a certain response), learning that the structure is a set of chunks involves a minimal role for fluency, whereas learning to detect symmetries involves a major role for fluency. Such a conjecture takes into account the role of fluency in symmetry processing (Reber et al., 1998) yet the minimal role fluency plays in standard AGL (e.g., Scott and Dienes, 2010a,b).

The conjecture is partly motivated by the proposal of Friederici et al. (2006) and Fitch and Friederici (2012) that a natural divide occurs for processing finite state or sub-finite state structures (such as chunks), on the one hand, and supra-finite state structures (such as symmetries), on the other. Friederici et al. (2006) compared learning an (AB)^n^ grammar (i.e., one in which AB can be repeated as many times as one likes, which can be produced by a finite state device) with learning a A^n^B^n^ grammar (i.e., one in which, however, many As there are, that many Bs must follow, which can only be generated from a supra-finite state device). Learning sorts of structures involved the operculum, but the latter in addition involved activation in Broca’s area. Thus, Broca’s area may be distinctively involved in learning supra-finite state structure [see Fitch and Friederici (2012) for a review of arguments and counter-arguments].

### Overview of the Current Research

Given the conjecture that fluency plays a key role in implicitly learning to detect symmetries but no role in implicitly learning to detect chunks (in conditions that encourage learning to make judgments rather than motor responses), we predict that the tonal symmetry in the materials developed by Jiang et al. (2012) will lead to processing fluency; but when chunk strength is used to define grammaticality in otherwise the same paradigm, fluency will play little to no role.

Following Jiang et al. (2012), in our materials the symmetry was defined over the tones with which Chinese syllables were spoken. There are four tones in Chinese (1–4) indicating flat, rising, falling-rising and falling phonetic characteristics in pitch, respectively. Tones 1 and 2 are traditionally categorized into ping (level) tones, while tones 3 and 4 are categorized into ze (oblique) tones. Like Jiang et al. (2012) we presented participants with strings of 10 syllables where the tone types of the first five syllables of a string predicted those of the last five by an inversion relation (**Figure 1**), e.g., if the tone type of the first syllable was ping, then the tone type of the sixth syllable was ze, and if the tone type of the second syllable was ping, then the tone type of the seventh syllable was ze, and so on. In the training phase, Chinese participants repeated back a number of “poems” instantiating this regularity; in the test phase they responded to new poems either following or violating the same rules of construction as the training items (with the required response varying across experiments). Chunks and repetition patterns were both controlled at the level of tones and tone types (ping/ze).

Experiments 1 and 2 used a RT task to investigate whether tonal symmetry produced fluency. In the training phase, participants listened to the sequences (as in Jiang et al., 2012). In the test phase, participants responded to each syllable of a string according to its tone (1/2/3/4) under the guise of a choice RT task. Experiment 2 aimed to replicate the results of Experiment 1 and compared the effect of implicit knowledge of grammaticality defined by chunk strength vs. symmetry on fluency. Training was twice as long as in Experiment 1 to explore the role of fluency in the acquisition of the different knowledge types over time (cf Opitz and Friederici, 2004, who found a shift to more abstract grammatical structures over time).

## Experiment 1

Experiment 1 aimed to show directly whether tonal symmetry produced processing fluency using a RT task. After an identical training phase as used in Jiang et al. (2012), participants entered the test phase in which they had to identify each tones (1–4) as quickly as they could. According to the effect of symmetry on fluency according to Reber et al. (1998) and the findings that people can implicit learning of the inversion symmetry when exposed to the inversion used by Jiang et al. (2012), people should respond to grammatical syllable strings faster than ungrammatical ones in the second half of the string (as the ping/ze structure of the second half is determined by the first half). If this prediction is supported, there will be evidence for a role of fluency in implicit learning.

### Method

#### Participants

Eighteen volunteers (14 female, aged 19–22, *M* = 22, *SD* = 2.68) from the university community participated in this experiment in exchange for credits or 30 RMB. None of the participants reported a history of hearing difficulties. Each participant was given informed consent before experiment. The study was approved by the Ethics Committee of the East China Normal University.

#### Design

This experiment used a two grammaticality (grammatical vs. ungrammatical) × 2 half (first vs. last) within-subject design. The dependent variables were error rate and response latency.

#### Materials

The symmetry relation used in this experiment was the same as Jiang et al. (2012), but the specific materials were different. In the present study, one syllable “you” was selected and displayed in each of the four tones, resulting in four tonal syllables: you1, you2, you3, and you4. You1 and you2 belong to the “ping” category, while you3 and you4 belong to “ze.” Each string consisted of 10 tonal syllables with the tone types (ping or ze) of the first five syllables predicting the tone types of the last half according to the relation of inversion: ping in the first half maps to ze in the second half in the same corresponding position in the sequence, and ze likewise maps to ping in corresponding positions, resulting to five ping-ze pairs (**Figure 1**).

Thirty-two grammatical tone type strings were generated according to the inversion rule, 16 of which were used as training strings, while the remaining served as test strings (see Supplementary Table S1). Each training tone type string was shown three times with different tonal syllables, resulting in 48 presentations of training tonal syllable strings in all. The test set comprised a combination of 16 grammatical tone type strings and 16 ungrammatical tone type strings which were created by violating the inversion rule in any two of five ping-ze pairs of 16 grammatical strings in the test. Each test tone type string was shown one time. None of the strings had a clear semantic interpretation.

Both repetition structure and chunking of grammatical and ungrammatical strings in the test were controlled. Repetition structure can be either local (Mathews and Roussel, 1997) or global (Vokey and Brooks, 1992). Local repetition structure reflects the similarity of a given element to that immediately preceding it; for example, the local repetition structure of “ping ping ze ze ping” or “ze ze ping ping ze” is 1010. The initial 1 represents the fact that the second tone type is the same as the first tone type; the following zero indicates that the third tone type is different than the second tone type and so forth. Global repetition structure reflects whether any element is the same as any other element in the string; for example, the global repetition structure of the same string is 11221. We calculated the global repetition proportion (GRP) and local repetition proportion (LRP) of the test tone-type strings based on the global repetition structures and local repetition structures, respectively. GRP is the maximum proportion of a test string’s global repetition structure that appeared in full (uninterrupted) in any of the training strings. LRP is the maximum proportion of the test string’s local repetition structure seen in training (Scott and Dienes, 2008). GRP and LRP were closely matched between grammatical and ungrammatical test tone-type strings (**Table 1**). Furthermore, none of the grammatical test strings had the same global or local repetition structures, in terms of tone types or tones 1–4, as any of the training strings.

**Table 1:** Mean LRP, GRP, SSP, RBP, RBPL, MFF, GACS, and AACS for grammatical and ungrammatical strings of Experiment 1 in terms of tone types *(M ± SD)*.

	Tone types	
	G	UG
LRP	0.54 ± 0.28	0.57 ± 0.07
GRP	0.59 ± 0.25	0.61 ± 0.06
SSP	0.59 ± 0.25	0.61 ± 0.06
RBP	0.54 ± 0.23	0.58 ± 0.08
RBPL	0.51 ± 0.23	0.48 ± 0.11
MFF	720.00 ± 0.00	720.00 ± 0.00
GACS	223.31 ± 5.73	223.17 ± 5.66
AACS	25.88 ± 2.32	26.16 ± 2.58

*G, grammatical; UG, ungrammatical; LRP, local repetition proportion; GRP, global repetition proportion; SSP, specific similarity proportion; RBP, repetition block structure proportion; RBPL, repetition block structure proportion length; MFF, mean feature frequency; GACS, global associative chunk strength; AACS, anchor associative chunk strength. Whether a difference in a property on a by-items test is significant between G and UG is not relevant to whether that difference could be used by participants to obtain a systematic difference in performance on G and UG items; nonetheless we note all differences are non-significant.*

In addition, specific similarity proportion (SSP), repetition block structure proportion (RBP), repetition block structure proportion length (RBPL), mean feature frequency (MFF), anchor associative chunk strength (AACS), and global associative chunk strength (GACS) were also closely matched between grammatical and ungrammatical test tone-type strings (**Table 1**). SSP was calculated the maximum proportion of the test string seen in full during training. RBS reflects the number of repetitions of consecutive elements in series; for example, the repetition block structure of “ping ping ze ze ping” or “ze ze ping ping ze” is 221. RBP is the maximum proportion of test strings’ repetition block structure occurring in a training string. RBPL is the maximum proportion of the test strings length for which the RBS is the same as one of the training strings. MFF was calculated for each tone-type string by averaging the number of times each tone type appeared in the training phase in each of the 10 positions (Jiang et al., 2012). Associative chunk strength (ACS) was defined as the frequency with which a chunk occurred in the training phase and here the chunk was defined as tone-type bigrams and trigrams. Then the GACS of each test tone-type string was calculated by averaging the above frequency scores across all of the chunks in the string. AACS was calculated the frequency with which tone-type bigrams and trigrams occurred in the beginning and ending positions (e.g., Knowlton and Squire, 1994; Kuhn and Dienes, 2005). In addition, the MFF, GACS and AACS in terms of tones 1–4 were also closely matched between grammatical and ungrammatical strings (see **Table 2**). The mean GACS for all test strings in terms of tones 1–4 were 56.66 (*SD* = 4.54). Given that only one syllable “you” was selected in this study, the structures of the tonal syllable strings were the same as that for the tones 1–4 strings (e.g., the tonal syllable strings was “you4 you3 you4 you2 you1- you2 you1 you1 you4 you3”; the tones 1–4 string was “43421- 21143”).

**Table 2:** Mean MFF, GACS, and AACS for grammatical and ungrammatical strings of Experiment 1 in terms of tones 1–4 *(M ± SD)*.

	Tones 1–4	
	G	UG
MFF	360.00 ± 0.00	360.00 ± 0.00
GACS	56.10 ± 4.35	57.22 ± 4.78
AACS	5.44 ± 2.13	5.72 ± 1.88

*G, grammatical; UG, ungrammatical; MFF, mean feature frequency; GACS, global associative chunk strength; AACS, anchor associative chunk strength. As for **Table 1**, we note the irrelevant fact that all differences are non-significant.*

Four tonal syllables (i.e., you1, you2, you3, you4) were created by Chinese pronunciation software (*Xunfei* interphonic 2.30, sampling rate = 22.05 kHz; so “you” is pronounced the Chinese and not the English way). Each of these syllables lasted for 300 ms and was spoken in a female voice. Each training tonal syllable string consisted of two half (i.e., the first half and second half) and 700 ms interval was interposed between each other to create a perceptual gap between the first half of the string and its inversion in the final half (cf. Mueller et al., 2010; Jiang et al., 2012). Each half consisted of five tonal syllables with a 100 ms interval between each other. Thus, each training tonal syllable string lasted for 4500 ms.

#### Procedure

In the training phase, participants were requested to listen to 144 trials in all, which consisted of three blocks of 48 grammatical tonal syllable strings presented in a random order in each block. In each trial, a warning tone was presented for 500 ms, followed by a 4500 ms tonal syllable string and a 5100 ms blank. Participants were instructed to listen to each tonal syllable string carefully and silently repeat it during the 5100 ms delay before the next trial. The training phase lasted about 27 min.

The test phase used a four-choice RT task. In this task, 32 test strings were presented in a random order. Each string consisted of 10 tonal syllables which were presented auditorily one by one through headphones. Each tonal syllable lasted for 0.3 s, and the participants were asked to press a corresponding key as quickly and accurately as possible according to the following assignment: the “D” key for “you1,” the “F” key for “you2,” the “J” key for “you3,” and the “K” key for “you4.” The maximum response time limit for each tonal syllable was set to 1700 ms. After a response-stimulus interval of 500 ms or 1100 ms (only after fifth syllable of each string), the next syllable was presented. Among strings was a 3000 s blank. Participants were required to respond to “D” and “F” with the middle and index finger of their left hand, and to “J” and “K” with the index and middle finger of their right hand.

### Results

Effects are tested using Bayes factors, *B*, to assess strength of evidence (e.g., Wagenmakers et al., in press); *p*-values are also reported so readers can in addition assess significance. A *B* of above three indicates substantial evidence for the alternative rather than the null hypothesis (Jeffreys, 1939) and below 1/3 substantial evidence for the null rather than alternative hypothesis. Thus, a *B* between 3 and 1/3 indicates data insensitivity for distinguishing the alternative and null hypotheses (see Dienes, 2014, 2015). *B*s testing condition differences are reported for differences justified by our predictions regarding the central issue of this paper, i.e., greater fluency for grammatical rather than non-grammatical stimuli. *B*_H(0,x)_ refers to a Bayes factor used to test the hypothesis that there is a difference between conditions, represented as a half-normal with a *SD* of x, against *H*_0_, the hypothesis of no difference (the “H” in *B*_H(0,x)_ stands for half-normal). Following Dienes (2014), when a roughly expected effect size can be specified, it is used as the *SD* of a half-normal. Qiao et al. (in preparation) varied ISI between tones with a difference of 40 ms; this difference induced a change in endorsement rates of 9% on average. Li et al. (2013) found training on similar material as the current experiment produced 12% more endorsements of grammatical rather than non-grammatical stimuli. That is, the difference in percentage endorsements is about the same. Thus, if symmetry detection was based entirely on fluency, grammatical and non-grammatical items would be expected to show an RT difference of 40 ms. As fluency is unlikely to be the only mechanism by which the strings are distinguished, 40 ms was taken as the rough maximum difference that could be expected, and the *SD* for the half-normal was set at half this value (i.e., at 20 ms), as recommended by Dienes (2014). This model of H1 was used for all inferential tests on RTs in the paper.

The average error rate was 3.9%; this was used as an estimate of the maximum plausible difference in error rate between conditions and H1 was modeled as a normal with a mean of zero and *SD* of 2% (i.e., about half of 3.9%). In this case, the Bayes factor is notated as *B*_N(0,2%)_ (where the N stands for a normal distribution, in this case with a mean of 0 and an *SD* of 2%).

#### Error Rates

**Table 3** shows mean error rates. A 2 (grammaticality: grammatical vs. ungrammatical) × 2 (half: first half vs. last half) repeated measures ANOVA on error rates was performed. This analysis showed insensitive evidence concerning the main effects [grammaticality: *F*(1,17) = 3.05, *p* = 0.10, $\eta_{p}^{2}$ = 1.52, *B*_N(0,2%)_ = 0.84; half: *F*(1,17) = 1.15, *p* = 0.30, $\eta_{p}^{2}$ = 0.06, *B*_N(0,2%)_ = 0.40] and interaction effects [*F*(1,17) = 2.46, *p* = 0.14, $\eta_{p}^{2}$ = 0.13, *B*_N(0,2%)_ = 1.16].

**Table 3:** Mean RT and error rates in each condition for Experiment 1 *(M ± SD)*.

Condition	Half 1		Half 2	
	G	UG	G	UG
RT (ms)	692 ± 72	687 ± 79	703 ± 61	713 ± 67
Error rates (%)	4.24 ± 2.85	4.10 ± 2.87	2.78 ± 1.64	4.51 ± 3.74

*G, grammatical; UG, ungrammatical.*

#### Response Times

For each participant, RTs with incorrect responses or which were more than three standard deviations from the mean RT were excluded from further analysis. Less than 6% of the data were omitted during this procedure in the experiment. The average RTs of valid trials across all the participants are illustrated in **Table 3**. A 2 (grammaticality: grammatical vs. ungrammatical) × 2 (half: first vs. last) repeated measures ANOVA showed insensitive evidence concerning the main effect of grammaticality [*F*(1,17) = 0.61, *p* = 0.45, $\eta_{p}^{2}$ = 0.04, *B*_H(0,20)_ = 0.39]. There was a main effect of half [*F*(1,17) = 12.67, *p* < 0.01, $\eta_{p}^{2}$ = 0.43, *B*_H(0,20)_ = 90.68] and an interaction [*F*(1,17) = 7.31, *p* = 0.02, $\eta_{p}^{2}$ = 0.30, *B*_H(0,20)_ = 10.58]. Simple effects between grammatical and ungrammatical sequences for each half separately, indicated that a difference between grammatical and ungrammatical sequences was not yet present in the first half [*t*(17) = 0.86, *p* = 0.40, *d* = 0.20, *B*_H(0,20)_ = 0.17] but was in the last half [*t*(17) = 2.29, *p* = 0.04, *d* = 0.54, *B*_H(0,20)_ = 3.92]. Further, to unpack the interaction in another way, simple effects between the first half and second half for the grammatical and ungrammatical strings separately were performed. The analyses revealed that there was only insensitive evidence for a difference between first half and second half for the grammatical strings[*t*(17) = 1.87, *p* = 0.08, *d* = 0.44, *B*_H(0,20)_ = 2.30] and strong evidence for a difference for the ungrammatical strings [*t*(17) = 4.42, *p* < 0.001, *d* = 1.04, *B*_H(0,20)_ = 1427.00].

### Discussion

Experiment 1 sought to investigate directly whether tonal symmetry produces processing fluency with a RT task, providing evidence for supporting the notion that fluency is a basis of expressing implicit knowledge in this paradigm. As predicted, reactions were faster for grammatical than ungrammatical strings in the second half of strings. However, Scott and Dienes (2010a,b) found no savings for the standard AGL paradigm using finite state grammars (cf also Seger, 1997). The apparent contrast of the current results with Scott and Dienes may be because different regularities were acquired in the two cases (e.g., symmetries vs. chunks), and the implicit knowledge in the two cases expresses itself differently; or it may be because of numerous other procedural differences, such as the use of visual vs. auditory stimuli and the consequent rather different way fluency was tested. Experiment 2 addressed this issue by exploring symmetry and chunk learning in the same experiment.

## Experiment 2

The aims of Experiment 2 were twofold. First, we sought to replicate the results of Experiment 1. Second, we sought to compare any fluency effects arising from chunk strength with those arising from symmetry. There is a large body of evidence that learning chunks is a key part of what people implicitly learn about structures (e.g., Dulany et al., 1984; Perruchet and Pacteau, 1990; Servan-Schreiber and Anderson, 1990; Pothos, 2007). Any knowledge of any regularity should in principle be able to express itself in faster processing of stimuli having that regularity, i.e., in fluency. However, just because knowledge might express itself one way, it does not mean it will, even on single process models (which allow independent noise in each mode of expression, so the effect can be made arbitrarily small for one mode of expression, Shanks and Berry, 2012).

In the Experiment 2, we orthogonally manipulated grammaticality and chunk strength for test strings in terms of chunks of tones 1–4 (see **Table 4**). Thus, there were four types of test strings: grammatical items of high chunk strength (GH), grammatical items of low chunk strength (GL), ungrammatical items of high chunk strength (UGH), and ungrammatical items of low chunk strength (UGL).

**Table 4:** Mean MFF, GACS, and AACS for grammatical and ungrammatical strings in terms of tones 1–4 (high chunk strength and low chunk strength; *M* ± *SD*).

Grammaticality	G		UG	
Chunks	H	L	H	L
MFF	360.00 ± 0.00	360.00 ± 0.00	360.00 ± 0.00	360.00 ± 0.00
GACS	65.77 ± 1.63	48.10 ± 1.75	64.69 ± 1.90	48.04 ± 1.44
AACS	5.81 ± 2.59	5.72 ± 2.19	6.00 ± 1.92	5.72 ± 2.00

*G, grammatical; UG, ungrammatical; H, high chunks; L, low chunks; MFF, mean feature frequency; GACS, global associative chunk strength; AACS, anchor associative chunk strength.*

### Method

#### Participants

Nineteen volunteers (11 female, aged 20–36, *M* = 25, *SD* = 5.27) from the university community participated in this experiment in exchange for credits or 30 RMB. All participants were given informed consent before experiment. None of the participants reported a history of hearing difficulties. The study was approved by the Ethics Committee of the East China Normal University. Data from one participant was discarded because of self-reported explicit knowledge of the inversion.

#### Design

This experiment used a 2 × 2 × 2 design, with grammaticality (grammatical vs. ungrammatical), half (first vs. last) and chunks (high vs. low) were the within-subjects factors. The dependent variables were error rates and response latency, as in Experiment 1.

#### Materials

The training tone type strings and test tone type strings used in Experiment 2 were identical with those used in Experiment 1 (see Supplementary Table S1). The training tonal syllables strings also were identical to those used in Experiment 1, while 32 new test tonal syllables strings were created to match the goal of the current experiment. Specifically, there were eight strings of each type (GH, GL, UGH, and UGL). **Table 4** shows that high chunk and low chunk strings differed substantially in GACS in terms of chunks of tones 1–4. In addition, the MFF, GACS, and AACS in terms of tones 1–4 were also closely matched between grammatical and ungrammatical strings.

#### Procedure

The procedure was the same as that of Experiment 1.

### Results

#### Error Rates

**Table 5** shows mean error rates. A 2 (grammaticality: grammatical vs. ungrammatical) × 2 (half: first half vs. last half) × 2 (chunks: high vs. low) repeated measures ANOVA on error rates showed insensitive evidence concerning the main effects [grammaticality: *F*(1,17) = 1.77, *p* = 0.20, $\eta_{p}^{2}$ = 0.09, *B*_N(0,2%)_ = 0.55; half: *F*(1,17) = 2.59, *p* = 0.13, $\eta_{p}^{2}$ = 0.13; *B*_N(0,2%)_ = 0.60; chunks: *F*(1,17) = 2.39, *p* = 0.14, $\eta_{p}^{2}$ = 0.12, *B*_N(0,2%)_ = 0.62] and interaction effects [grammaticality × half: *F*(1,17) = 0.34, *p* = 0.57, $\eta_{p}^{2}$ = 0.02, *B*_N(0,2%)_ = 0.51; chunks × half: *F*(1,17) = 0.31, *p* = 0.59, $\eta_{p}^{2}$ = 0.02, *B*_N(0,2%)_ = 0.52; grammaticality × chunks: *F*(1,17) = 1.10, *p* = 0.31, $\eta_{p}^{2}$ = 0.06, *B*_N(0,2%)_ = 0.65; half × grammaticality × chunks: *F*(1,17) = 0.03, *p* = 0.87, $\eta_{p}^{2}$ < 0.01, *B*_N(0,2%)_ = 0.68].

**Table 5:** Mean RT and error rates in each condition for Experiment 2 (*M* ± *SD*).

Condition			RT(ms)	Error rates (%)
Half 1	G	H	695 ± 93	4.31 ± 3.82
		L	708 ± 94	3.33 ± 3.09
	UG	H	701 ± 81	3.33 ± 3.93
		L	711 ± 98	3.47 ± 4.30
Half 2	G	H	713 ± 96	5.42 ± 4.79
		L	703 ± 102	4.03 ± 2.59
	UG	H	725 ± 91	4.03 ± 3.11
		L	718 ± 93	3.47 ± 2.99

*G, grammatical; UG, ungrammatical; H, high chunks; L, low chunks*.

#### Response Times

For each participant, RTs with incorrect responses or which were more than three standard deviations from the mean RT were excluded from further analysis. Less than 6% of the data were omitted during this procedure in the experiment. The average RTs of valid trials across all the participants are illustrated in **Table 5**. A 2 (grammaticality: grammatical vs. ungrammatical) × 2 (half: first vs. last) × 2 (chunks: high vs. low) repeated measures ANOVA showed insensitive evidence concerning the main effect of grammaticality [*F*(1,17) = 2.74, *p* = 0.12, $\eta_{p}^{2}$ = 0.14, *B*_H(0,20)_ = 1.57] and chunks [*F*(1,17) = 0.08, *p* = 0.79, $\eta_{p}^{2}$ < 0.01, *B*_H(0,20)_ = 0.44], and evidence for a main effect of half [*F*(1,17) = 4.56, *p* = 0.048, $\eta_{p}^{2}$ = 0.21, *B*_H(0,20)_ = 3.31]. Further, there was evidence for interaction effects between half and grammaticality [*F*(1,17) = 5.04, *p* = 0.04, $\eta_{p}^{2}$ = 0.23, *B*_H(0,20)_ = 3.11] and between half and chunks [*F*(1,17) = 8.37, *p* = 0.01, $\eta_{p}^{2}$ = 0.33, *B*_N(0,20)_ = 8.56]. This analysis also showed insensitive evidence concerning the interaction of grammaticality and chunks [*F*(1,17) = 0.004, *p* = 0.95, $\eta_{p}^{2}$ < 0.001, *B*_N(0,20)_ = 0.67] and the half × grammaticality × chunks interaction (*F*(1,17) = 0.17, *p* = 0.69, $\eta_{p}^{2}$ = 0.10, *B*_N(0,20)_ = 0.65].

To interpret the interaction of grammaticality and half, simple effects between grammatical and ungrammatical sequences for each half separately were performed, The results showed that there was substantial evidence for a difference between grammatical strings and ungrammatical strings in the last half [*t* = 2.23, *p* = 0.04, *d* = 0.52, *B*_H(0,20)_ = 4.20], and insensitive evidence for a difference in the first half [*t* = 0.81, *p* = 0.43, *d* = 0.19, *B*_H(0,20)_ = 0.62]. Further, simple effects between the first half and second half for the grammatical and ungrammatical strings separately were performed. The analyses indicated there was only insensitive evidence for a difference between first half and second half for the grammatical strings [*t* = 1.16, *p* = 0.26, *d* = 0.27, *B*_H(0,20)_ = 0.89] and strong evidence for a difference for the ungrammatical strings (*t* = 2.79, *p* = 0.01, *d* = 0.66, *B*_H(0,20)_ = 12.72].

To interpret the interaction of chunks and half, simple effects between high chunks and low chunks strings for each half separately were performed, The analyses indicated that there was no difference between high and low chunks strings in the last half [*t*(17) = 1.19, *p* = 0.25, *d* = 0.28, *B*_H(0,20)_ = 0.17] and insensitive evidence for whether or not there was a difference in the first half [*t*(17) = 1.86, *p* = 0.08, *d* = 0.44, *B*_H(0,20)_ = 2.26]. Further, simple effects between the first half and second half for the high and low chunks strings separately were performed. The analyses revealed that there was only insensitive evidence for a difference between first half and second half for the low chunks strings [*t*(17) = 0.23, *p* = 0.82, *d* = 0.05, *B*_N(0,20)_ = 0.43] and strong evidence for a difference for the high chunks strings [*t*(17) = 3.15, *p* = 0.006, *d* = 0.74, *B*_N(0,20)_ = 15.75].

In addition, we compared the last half of low chunk strength grammatical items with that of high chunk strength ungrammatical items which maximally contrast symmetry vs. chunk knowledge (Forkstam et al., 2006; Petersson et al., 2012). This analysis showed that shorter RTs for the low chunk strength grammatical items than for high chunk strength ungrammatical items, *t*(17) = 2.23, *p* = 0.04, *d* = 0.53, *B*_H(0,20)_ = 4.93. Taken together, these results suggest that grammaticality status independent of ACS produces processing fluency in the four-choice RT task.

### Discussion

Experiment 2 revealed that symmetry sped up responding to the second half of strings, indicating a fluency effect, replicating findings of Experiment 1. Experiment 2 investigated in addition whether such an effect behaved differently for symmetry and chunk strength. Indeed, there was weak evidence that chunk strength produced a fluency effect, albeit one that expressed itself especially in the first half of each string. Thus, speeded responding was guided by different information in the different halves of the string. The second half of the string was the only half in which symmetry could be used; and in this half symmetry was stronger than chunk strength in guiding responses. Conversely, in the first half, where symmetry could play no rule, chunk strength may have guided responses.

Symmetry might have controlled responding in the second half of strings because there was no chunk strength differences to do so; but **Table 6** indicates that this is not so. The degree of chunk strength difference was virtually the same in each half. Thus, symmetry overrides the use of chunk strength even when chunk strength is highly informative.

**Table 6:** Mean GACS for the first half and second half of strings in terms of tones 1–4 (high chunk strength and low chunk strength; *M* ± *SD*).

Half	Half 1		Half 2	
Chunks	H	L	H	L
GACS	58.98 ± 7.37	40.20 ± 8.60	55.80 ± 7.96	41.30 ± 7.87

*GACS, global associative chunk strength; H, high chunks; L, low chunks.*

The pattern indicates a difference in the way chunk strength and symmetry were used. This difference in itself need not indicate that chunk strength and symmetry are processed by fundamentally different computational mechanisms. Both types of information may express themselves as fluency in a coordinated way. One could in principle construct a single model that used structures (symmetry vs. chunks) according to their predictive power (so e.g., symmetry dominated predictions just when it was actually predictive). Alternatively, one could construct two mechanisms that competed for predicting outputs, producing the same effect of symmetry and chunks controlling output at different times. Nonetheless, the simplest model that jointly processed chunks and symmetry would allow each to influence responding. But what we found was that where both structures were relevant (in the second half), only symmetry produced fluency. In the first half, where only chunks were relevant, the evidence for fluency effects was weak.

## General Discussion

The purposes of the current research were twofold. First, we sought to establish whether tonal symmetry produces processing fluency. Second, we sought to explore whether symmetry and chunk strength express themselves differently in fluency, as an indication of different mechanisms being involved for sub- and supra-finite state processing. We found that symmetry could be used to speed responding to identifying the stimulus displayed; i.e., symmetry was expressed as a fluency effect. We found that by our paradigm, chunk strength also facilitated responding to identifying the stimulus, as is found for example in the SRT task (Janacsek et al., 2014; Sanchez et al., 2015), but was not found by Scott and Dienes (2010a,b) for the AGL task. We now consider explanations for this pattern of findings.

The results found evidence for symmetry learning even when alternative structures that people could learn (like repetition structures) are carefully controlled with new materials. Further, such knowledge expresses itself in fluency, consistent with the proposal that symmetry is useful for reducing processing. The claim that symmetry reduces the need for processing is not as self-evident for sequential rather than simultaneous presentation of the symmetry structure (contrast the different computational mechanisms needed for processing symmetry in the language and visual domains, Tyler, 2002). Despite the greater computational complexity needed for processing symmetry in the sequential rather than simultaneous case, people still exploit symmetry in tone sequences.

The finding that fluency is a consequence of the implicit learning of symmetry, is consistent with the argument that fluency can be a reason that people prefer symmetry in many contexts (Reber, 2003; Reber et al., 2004). Scott and Dienes (2010a) found that when the structures were chunks, people used fluency as a last resort; in that sense, fluency could be regarded as (to use Phil Higham’s phrase, cited in Dienes and Scott) a “dumb heuristic” i.e., influencing responding only in the absence of actual implicit knowledge of chunks. When it comes to symmetry, fluency expresses actual structural knowledge and thus is more than a dumb heuristic (Scott and Dienes, 2010a). Further, in Scott and Dienes, actual chunk knowledge did not express itself as fluency. This contrast appears to indicate a difference between fluency and chunk strength and motivated our Experiment 2. We found that chunk strength and symmetry did express themselves differently in fluency. In the second half of the string, where symmetry provides predictive power, only symmetry produced fluency and not chunks. In the first half of the string, chunks may have produced fluency effects. Thus, the theoretical contrast between different regions and mechanisms processing sub- and supra-finite state processing is supported by the current results (Fitch and Friederici, 2012).

A question remains why Scott and Dienes (2010a) did not find fluency effects for chunks whereas we find possible effects here. One conjecture is that in the Scott and Dienes (2010a) case a single response was used to indicate that the participant had identified the whole string. In the current study, one response was used to indicate the identification of a component of a string (as in the SRT case). Thus, in the current case knowledge of structure could come to control the sequence of motor responses. Future research should compare symmetry and chunk structure learning where a single response is used to indicate the identification of the whole string in order to measure perceptual fluency without the chance for motor learning to play a role.

One limitation of the current study is that the implicitness of the knowledge was not independently established. Jiang et al. (2012) and Li et al. (2013) using very similar materials found largely unconscious knowledge of the structures, and we have relied on this previous work for describing the knowledge as implicit in the current study. However, there were differences between the procedures. For example, the current study used a RT task and the previous work used a classification task; this difference is not likely to increase the amount of conscious knowledge in itself. On the other hand, the current study used a single syllable (“you”) whereas past studies used different syllables; the richness of irrelevant information in previous studies may have favored the development of unconscious knowledge of the relevant structure in those studies (cf Norman et al., 2011). Furthermore, it leaves open the question whether we might obtain the similar results, if we had used different syllables instead of the single syllable in the study. It’s worth noting that, in the current study, both symmetry relation and chunks were defined over tones and were syllable-independent. This, in combined with the results of Jiang et al. (2012) and Li et al. (2013), which used a variety of syllables, suggested that similar results should obtain even with different syllables. Future research can explore this point.

Natural languages contain some structures above finite-state (e.g., phrase structure grammars) which uniquely produce various symmetries (cf. Chomsky, 1959). Our study shows that adult implicit learning mechanisms can acquire such structures. These results thus allow the possibility for the same or similar mechanisms underlying implicit learning in adults to have been involved in the child acquisition of language (Reber, 1993). Whatever the case may be in that respect, our study shows that adult implicit learning poses problems for computational models that depend on chunk learning (cf. Rohrmeier and Cross, 2014); what type of computational model could explain the results remains an open question.

In sum, we found that people could learn symmetry in sequential tone structures; and that this knowledge expresses itself as fluency. The symmetries of the supra-finite state structures of language, even when not consciously noticed, may modulate the flow of comprehension, even over-riding simple sequential dependencies.

## Author Contributions

XG, FL, XL, QQ, and ZD designed research. XL and QQ performed research. XG, XL, and ZD analyzed data and joined in the interpretation of data. ZD, XL, FL, and XG wrote the paper. XL and FL contributed equally to this work.

## Conflict of Interest Statement

The authors declare that the research was conducted in the absence of any commercial or financial relationships that could be construed as a potential conflict of interest.
