# Supplementary material for: Fluency Expresses Implicit Knowledge of Tonal Symmetry
Source: Front Psychol. 2016 Feb 3;7:57. doi: 10.3389/fpsyg.2016.00057 (PMC4737865; doi:10.3389/fpsyg.2016.00057)
Supplement: Supplementary file 1 [file Data_Sheet_1.DOCX]

***Supplementary Material***

**Fluency Expresses Implicit Knowledge of Tonal Symmetry**

Xiaoli Ling, Fengying Li, Fuqiang Qiao, Xiuyan Guo* and Zoltan Dienes

*** Correspondence:** Xiuyan Guo: [wlkc_xyguo@126.com](mailto:wlkc_xyguo@126.com)

**Supplementary Table 1. Training and test tone type strings used in the experiment 1 and 2.**

| Sort | Inversion |
| --- | --- |
| 1 | p p p p p - z z z z z |
| 1 | p p p p z - z z z z p |
| 1 | p p p z z - z z z p p |
| 1 | p p z z z - z z p p p |
| 1 | p z p p z - z p z z p |
| 1 | p z p z p - z p z p z |
| 1 | p z z p z - z p p z p |
| 1 | p z z z z - z p p p p |
| 1 | z p p p p - p z z z z |
| 1 | z p p z p - p z z p z |
| 1 | z p z p z - p z p z p |
| 1 | z p z z p - p z p p z |
| 1 | z z p p p - p p z z z |
| 1 | z z z p p - p p p z z |
| 1 | z z z z p - p p p p z |
| 1 | z z z z z - p p p p p |
| 2 | z p z p p - p z p z z |
| 2 | p p z p z - z z p z p |
| 2 | p p z p p - z z p z z |
| 2 | z z p z z - p p z p p |
| 2 | p p p z p - z z z p z |
| 2 | p z p p p - z p z z z |
| 2 | z z p z p - p p z p z |
| 2 | p z p z z - z p z p p |
| 2 | z p z z z - p z p p p |
| 2 | z p p z z - p z z p p |
| 2 | z z z p z - p p p z p |
| 2 | p z z p p - z p p z z |
| 2 | z z p p z - p p z z p |
| 2 | p p z z p - z z p p z |
| 2 | z p p p z - p z z z p |
| 2 | p z z z p - z p p p z |
| 3 | p p z p z - p z z z p |
| 3 | z p p z z - z p z p p |
| 3 | p z p z z - p p z z p |
| 3 | p p z z p - z p p z z |
| 3 | z z p p z - p z z p p |
| 3 | z z z p z - p z p p p |
| 3 | z z p z p - z p p p z |
| 3 | z p z p p - z z p p z |
| 3 | p p z p p - z p z z z |
| 3 | z z p z z - p z p p p |
| 3 | p z z p p - p z p z z |
| 3 | z p p p z - z z p z p |
| 3 | p z p p p - z z p z z |
| 3 | z p z z z - p p z p p |
| 3 | p p p z p - z p z z z |
| 3 | p z z z p - p p z p z |

*Note.* p = ping, z = ze; 1 = grammatical tone type strings in the training phase, 2 = grammatical tone type strings in the test phase, and 3 = ungrammatical tone type strings in the test phase.
